# Bis[tris­(1,10-phenanthroline)nickel(II)] tris­[dicyanidoargentate(I)] nitrate 4.2-hydrate

**DOI:** 10.1107/S1600536808031413

**Published:** 2008-10-09

**Authors:** Muhammad Monim-ul-Mehboob, Muhammad Tufail, Muhammad Altaf, Helen Stoeckli-Evans, Saeed Ahmad, Syed Hassan Iftikhar

**Affiliations:** a Department of Chemistry, University of Engineering and Technology, Lahore 54890, Pakistan; b Institute of Physics, University of Neuchâtel, rue Emile-Argand 11, CH-2009 Neuchâtel, Switzerland

## Abstract

The title compound, [Ni(C_12_H_8_N_2_)_3_]_2_[Ag(CN)_2_]_3_(NO_3_)·4.2H_2_O, crystallizes with two independent [Ni(phen)_3_]^2+^ cations (phen is 1,10-phenanthroline; both Ni atoms have threefold symmetry and N_6_ donor sets), three near-linear [Ag(CN)_2_]^−^ anions, one nitrate anion (N site symmetry 3) and 4.2 water mol­ecules of crystallization, some of which are disordered. The [Ag(CN)_2_]^−^ anions are situated within cavities created by the phenanthroline ligands of adjacent [Ni(phen)_3_]^2+^ cations. Some short C—H⋯O and C—H⋯N inter­actions may help to establish the packing.

## Related literature

For a closely related structure containing 2,2′-bipyridine, see: Černák *et al.* (1994 ▶). For related literature, see: Ahmad *et al.* (2007 ▶); Allen (2002 ▶); Ren *et al.* (2005 ▶); Sastri *et al.* (2003 ▶); Shorrock *et al.* (2002 ▶); Zhang *et al.* (2006 ▶).
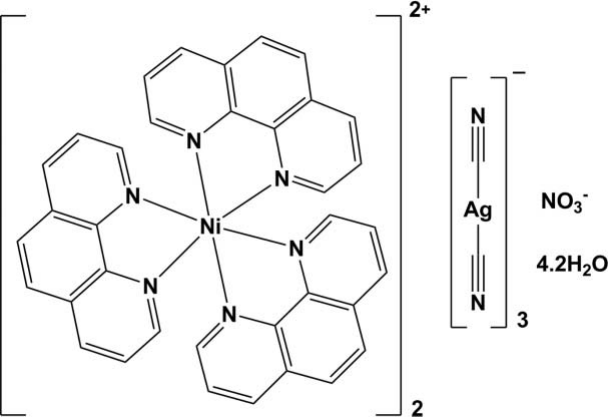


## Experimental

### 

#### Crystal data

[Ni(C_12_H_8_N_2_)_3_]_2_[Ag(CN)_2_]_3_(NO_3_)·4.2H_2_O
*M* *_r_* = 1816.05
Trigonal, 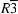

*a* = 16.2738 (7) Å
*c* = 46.398 (2) Å
*V* = 10641.6 (8) Å^3^
*Z* = 6
Mo *K*α radiation
μ = 1.41 mm^−1^
*T* = 173 (2) K
0.50 × 0.40 × 0.30 mm

#### Data collection

Stoe IPDSII diffractometer
Absorption correction: multi-scan (*MULscanABS* in *PLATON*; Spek, 2003 ▶) *T*_min_ = 0.454, *T*_max_ = 0.651
28787 measured reflections
6400 independent reflections
5514 reflections with *I* > 2σ(*I*)
*R*_int_ = 0.031

#### Refinement

*R*[*F* ^2^ > 2σ(*F* ^2^)] = 0.031
*wR*(*F* ^2^) = 0.086
*S* = 1.03
6400 reflections
353 parameters
H-atom parameters constrained
Δρ_max_ = 0.89 e Å^−3^
Δρ_min_ = −0.80 e Å^−3^

### 

Data collection: *X-AREA* (Stoe & Cie, 2006 ▶); cell refinement: *X-AREA*; data reduction: *X-RED32* (Stoe & Cie, 2006 ▶); program(s) used to solve structure: *SHELXS97* (Sheldrick, 2008 ▶); program(s) used to refine structure: *SHELXL97* (Sheldrick, 2008 ▶); molecular graphics: *PLATON* (Spek, 2003 ▶) and *Mercury* (Macrae *et al.*, 2006 ▶); software used to prepare material for publication: *SHELXL97*.

## Supplementary Material

Crystal structure: contains datablocks I, global. DOI: 10.1107/S1600536808031413/hb2786sup1.cif

Structure factors: contains datablocks I. DOI: 10.1107/S1600536808031413/hb2786Isup2.hkl

Additional supplementary materials:  crystallographic information; 3D view; checkCIF report

## Figures and Tables

**Table 1:** Selected bond lengths (Å)

Ag1—C25	2.043 (3)
Ag1—C26	2.055 (3)
Ni1—N1	2.0903 (17)
Ni1—N2	2.1014 (18)
Ni2—N4	2.0898 (16)
Ni2—N3	2.0925 (15)

**Table 2:** Hydrogen-bond geometry (Å, °)

*D*—H⋯*A*	*D*—H	H⋯*A*	*D*⋯*A*	*D*—H⋯*A*
C3—H3⋯N5^i^	0.95	2.45	3.284 (4)	147
C5—H5⋯O1^ii^	0.95	2.36	3.176 (5)	144
C8—H8⋯O1*WA*^iii^	0.95	2.54	3.465 (4)	166
C17—H17⋯O1	0.95	2.47	3.423 (4)	177
C20—H20⋯N6^iii^	0.95	2.60	3.312 (3)	132

Symmetry codes: (i) 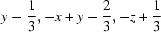; (ii) 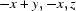; (iii) 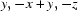.

**Fig. 1.**
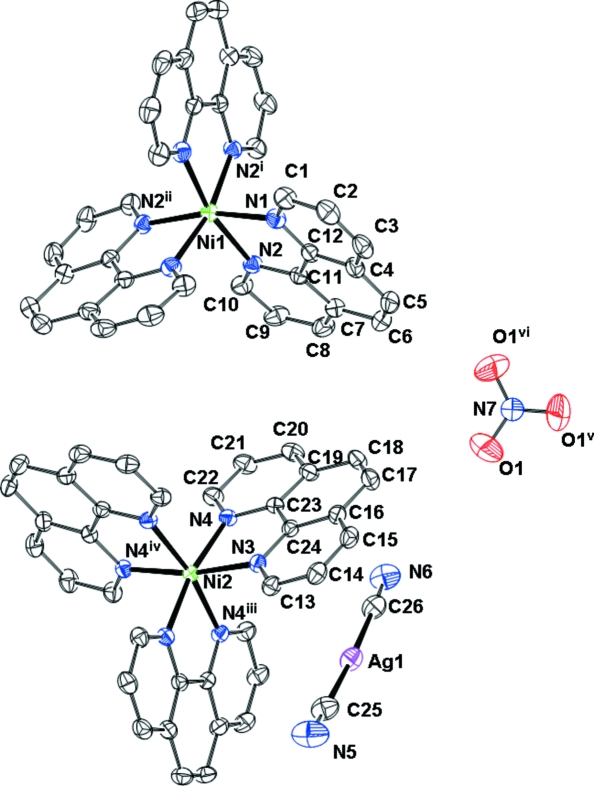
The molecular structure of (I), showing displacement ellipsoids drawn at the 50% probability level [H atoms and water molecules of crystallization have been omitted for clarity; Symmetry codes: (i) -y + 1, x-y, z; (ii) -x + y+1,-x + 1, z; (iv) -y + 1, x-y + 1, z; (iii) -x + y, -x + 1, z;(v) -y, x-y, z; (vi) -x + y, -x, z]

**Fig. 2.**
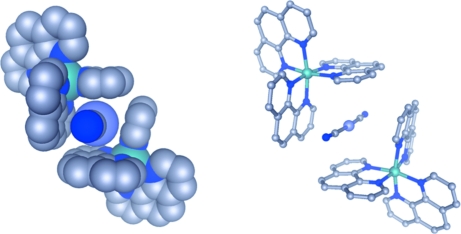
Two views of the [Ag(CN)2]- anion situated within a cavity created by the phenanthroline ligands of two [Ni(phen)3]2+ cations.

**Fig. 3.**
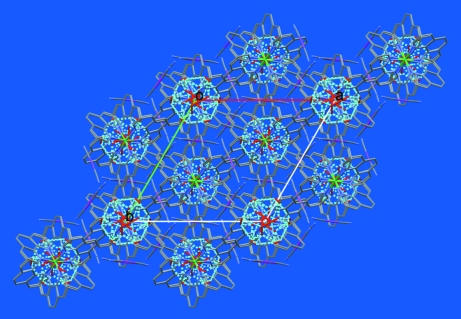
A view along the c axis of the crystal packing of (I) showing the formation of the hexagonally shaped water clusters [The H-atoms have been omitted for clarity; the O···O contacts are shown as blue dotted lines].

**Fig. 4.**
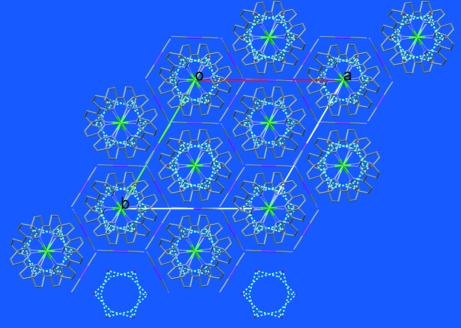
A view along the c axis of the crystal packing of (II) [The published coordinates have been transformed from rhombohedral to hexagonal axes; the H-atoms have been removed for clarity, and the O···O contacts are shown as blue dotted lines].

## supplementary crystallographic information

### Comment

Supramolecular structures based on [Ag(CN)_2_]^-^ anions are of significant interest because of their potential for structural, magnetic and catalytic applications, as witnessed by some recent work in this area (Ahmad *et al.*, 2007; Ren *et al.*, 2005; Shorrock *et al.*, 2002; Zhang *et al.*, 2006). We have begun investigations of the structural and chemical properties of metal(II)—Ag(I) coordination polymers that contain the [Ag(CN)_2_]^-^ anion as a bridging unit (Ahmad *et al.*, 2007). Mixed-ligand metal complexes of 1,10-phenanthroline (phen), and its substituted derivatives, are also interesting because they play an important role in biological systems, such as binding small molecules to DNA (Sastri *et al.*,  2003). A search of the Cambridge Structural Database (CSD V5.29, last update Jan 2008; Allen, 2002) revealed the presence of more than 50 complexes involving the [Ni(phen)_3_]^2+^ cation. In the present study we attempted to prepare a coordination polymer consisting of [Ni(phen)_2_]^2+^ cations and [Ag(CN)_2_]^-^, but instead, the title compound, (I), was isolated.

The molecular structure of (I) is shown in Fig. 1. The two independent [Ni(phen)_3_]^2+^ cations have 3-fold symmetry and both Ni atoms have octahedral environments formed by six nitrogen atoms from three 1,10-phenanthroline ligands (Table 1) with normal bond distances and angles. The coordination environment of metal in the [Ag(CN)_2_]^-^ anion is close to linear [C—Ag—C = 176.78 (13)°].

In the crystal structure of (I), the [Ag(CN)_2_]^-^ anions are situated within cavities created by the phenanthroline ligands of two [Ni(phen)_3_]^2+^ cations (see Fig. 2), hence the silver atoms are isolated from one another. The cationic and anionic units are associated with each other through C—H···O and C—H···N weak interactions (Table 2). The disordered water molecules of crystallization in (I) occupy regions in the vicinity of the 3 symmetry positions (Fig. 3).

The crystal structure of (I) is very similar to that of bis[tris(bipyridine)nickel(II)] tris[dicyanoargentate(I)] chloride nonahydrate, (II), (Černák *et al.*, 1994). Both crystallize in the trigonal space group *R*3, with a similar disposition in the crystal of the [Ni(phen)_3_]^2+^ and [Ag(CN)_2_]^-^ ionic moeties.  In (II), however, the secondary anion, Cl^-^, is partially distributed over the positions of the water molecules of crystallization (Fig. 4).

### Experimental

An aqueous mixture of Ni(NO_3_)_2_^.^6H_2_O, 1,10-phenanthroline (phen) and K[Ag(CN)_2_], was made up in a molar ratio of 1:1:2. After stirring the mixture for 25–30 min, a pink precipitate appeared. This was filtered off and the colourless filtrate left to evaporate slowly at room temperature. After a few days pink blocks of (I) were obtained, which were washed with methanol.

### Refinement

The aromatic H-atoms were included in calculated positions and treated as riding atoms: C—H = 0.95 Å with *U*_iso_(H) = 1.2*U*_eq_(C). The H atoms of the water molecules could not be located.

### Crystal data

**Table:**

[Ni(C_12_H_8_N_2_)_3_]_2_[Ag(CN)_2_]_3_(NO_3_)·4.2H_2_O	*D*_x_ = 1.700 Mg m^−^^3^
*M**_r_* = 1816.05	Mo *K*α radiation, λ = 0.71073 Å
Trigonal, *R*3	Cell parameters from 29311 reflections
Hall symbol: -R 3	θ = 1.7–29.6°
*a* = 16.2738 (7) Å	µ = 1.41 mm^−^^1^
*c* = 46.398 (2) Å	*T* = 173 K
*V* = 10641.6 (8) Å^3^	Block, pink
*Z* = 6	0.50 × 0.40 × 0.30 mm
*F*(000) = 5472	

### Data collection

**Table:**

Stoe IPDSII diffractometer	6400 independent reflections
Radiation source: fine-focus sealed tube	5514 reflections with *I* > 2σ(*I*)
graphite	*R*_int_ = 0.031
φ and ω scans	θ_max_ = 29.2°, θ_min_ = 1.7°
Absorption correction: multi-scan (MULscanABS in PLATON; Spek, 2003)	*h* = −21→20
*T*_min_ = 0.454, *T*_max_ = 0.651	*k* = −19→22
28787 measured reflections	*l* = −63→63

### Refinement

**Table:**

Refinement on *F*^2^	Secondary atom site location: difference Fourier map
Least-squares matrix: full	Hydrogen site location: inferred from neighbouring sites
*R*[*F*^2^ > 2σ(*F*^2^)] = 0.031	H-atom parameters constrained
*wR*(*F*^2^) = 0.086	*w* = 1/[σ^2^(*F*_o_^2^) + (0.0528*P*)^2^ + 11.6906*P*] where *P* = (*F*_o_^2^ + 2*F*_c_^2^)/3
*S* = 1.03	(Δ/σ)_max_ = 0.001
6400 reflections	Δρ_max_ = 0.89 e Å^−^^3^
353 parameters	Δρ_min_ = −0.80 e Å^−^^3^
0 restraints	Extinction correction: SHELXL97 (Sheldrick, 2008), Fc^*^=kFc[1+0.001xFc^2^λ^3^/sin(2θ)]^-1/4^
Primary atom site location: structure-invariant direct methods	Extinction coefficient: 0.00026 (3)

### Special details

**Table:**

Geometry. Bond distances, angles *etc*. have been calculated using the rounded fractional coordinates. All su's are estimated from the variances of the (full) variance-covariance matrix. The cell e.s.d.'s are taken into account in the estimation of distances, angles and torsion angles
Refinement. Refinement of *F*^2^ against ALL reflections. The weighted *R*-factor *wR* and goodness of fit *S* are based on *F*^2^, conventional *R*-factors *R* are based on *F*, with *F* set to zero for negative *F*^2^. The threshold expression of *F*^2^ > σ(*F*^2^) is used only for calculating *R*-factors(gt) *etc*. and is not relevant to the choice of reflections for refinement. *R*-factors based on *F*^2^ are statistically about twice as large as those based on *F*, and *R*- factors based on ALL data will be even larger.

### Fractional atomic coordinates and isotropic or equivalent isotropic displacement parameters (Å^2^)

**Table:**

	*x*	*y*	*z*	*U*_iso_*/*U*_eq_	Occ. (<1)
Ni1	0.66667	0.33333	0.09760 (1)	0.0219 (1)	
N1	0.55774 (12)	0.22921 (12)	0.12273 (3)	0.0263 (4)	
N2	0.54772 (11)	0.30552 (11)	0.07265 (3)	0.0236 (4)	
C1	0.56354 (16)	0.19654 (16)	0.14863 (4)	0.0331 (6)	
C2	0.48349 (18)	0.13805 (16)	0.16522 (5)	0.0366 (6)	
C3	0.39524 (17)	0.10971 (15)	0.15449 (5)	0.0343 (6)	
C4	0.38610 (15)	0.13989 (14)	0.12682 (4)	0.0293 (5)	
C5	0.29696 (16)	0.11155 (16)	0.11340 (5)	0.0359 (6)	
C6	0.29168 (15)	0.14076 (16)	0.08686 (5)	0.0358 (6)	
C7	0.37639 (14)	0.20643 (14)	0.07144 (4)	0.0284 (5)	
C8	0.37537 (15)	0.24171 (16)	0.04399 (5)	0.0338 (6)	
C9	0.45898 (16)	0.30945 (17)	0.03200 (4)	0.0339 (6)	
C10	0.54358 (15)	0.34083 (15)	0.04711 (4)	0.0290 (5)	
C11	0.46468 (13)	0.23886 (13)	0.08459 (4)	0.0235 (5)	
C12	0.47022 (14)	0.20149 (13)	0.11212 (4)	0.0242 (5)	
Ni2	0.33333	0.66667	0.08188 (1)	0.0186 (1)	
N3	0.23944 (10)	0.54770 (11)	0.10601 (3)	0.0211 (4)	
N4	0.33131 (11)	0.55851 (11)	0.05706 (3)	0.0211 (4)	
C13	0.19556 (13)	0.54382 (14)	0.13060 (4)	0.0254 (5)	
C14	0.13796 (14)	0.45900 (15)	0.14521 (4)	0.0294 (5)	
C15	0.12376 (14)	0.37531 (14)	0.13348 (4)	0.0285 (5)	
C16	0.16541 (13)	0.37618 (14)	0.10678 (4)	0.0253 (5)	
C17	0.14959 (15)	0.29206 (14)	0.09207 (4)	0.0302 (6)	
C18	0.18904 (15)	0.29717 (14)	0.06616 (4)	0.0298 (5)	
C19	0.25180 (14)	0.38706 (13)	0.05303 (4)	0.0253 (5)	
C20	0.29664 (16)	0.39689 (15)	0.02637 (4)	0.0297 (6)	
C21	0.35881 (16)	0.48563 (15)	0.01624 (4)	0.0307 (6)	
C22	0.37553 (14)	0.56505 (14)	0.03227 (4)	0.0269 (5)	
C23	0.27092 (12)	0.47077 (12)	0.06744 (4)	0.0205 (4)	
C24	0.22410 (12)	0.46493 (12)	0.09427 (4)	0.0207 (4)	
O2W	0.043 (2)	0.0466 (16)	0.0515 (3)	0.128 (12)	0.200
O3W	0.1111 (11)	0.0799 (8)	0.0346 (3)	0.080 (5)	0.200
Ag1	−0.00155 (1)	0.47423 (1)	0.08079 (1)	0.0352 (1)	
N5	−0.0135 (2)	0.6044 (2)	0.12942 (5)	0.0638 (10)	
N6	0.02865 (18)	0.34982 (19)	0.03319 (4)	0.0486 (7)	
C25	−0.01043 (19)	0.5588 (2)	0.11172 (5)	0.0432 (8)	
C26	0.01549 (17)	0.39317 (18)	0.05002 (5)	0.0370 (7)	
O1	0.0012 (2)	0.07445 (19)	0.12175 (8)	0.0961 (13)	
N7	0.00000	0.00000	0.12009 (8)	0.0415 (8)	
O1WA	0.0227 (2)	0.1884 (2)	0.00412 (7)	0.0713 (11)	0.850
O1WB	−0.0212 (12)	0.1577 (12)	0.0217 (4)	0.067 (6)	0.150
H1	0.62440	0.21370	0.15610	0.0400*	
H2	0.49050	0.11820	0.18390	0.0440*	
H3	0.34040	0.07000	0.16560	0.0410*	
H5	0.24010	0.07100	0.12350	0.0430*	
H6	0.23140	0.11800	0.07810	0.0430*	
H8	0.31730	0.21890	0.03380	0.0410*	
H9	0.45930	0.33490	0.01350	0.0410*	
H10	0.60070	0.38950	0.03880	0.0350*	
H13	0.20380	0.60120	0.13860	0.0300*	
H14	0.10900	0.45910	0.16300	0.0350*	
H15	0.08600	0.31710	0.14330	0.0340*	
H17	0.11070	0.23180	0.10070	0.0360*	
H18	0.17510	0.24030	0.05640	0.0360*	
H20	0.28390	0.34230	0.01550	0.0360*	
H21	0.39040	0.49320	−0.00160	0.0370*	
H22	0.42020	0.62620	0.02520	0.0320*	

### Atomic displacement parameters (Å^2^)

**Table:**

	*U*^11^	*U*^22^	*U*^33^	*U*^12^	*U*^13^	*U*^23^
Ni1	0.0225 (1)	0.0225 (1)	0.0209 (2)	0.0112 (1)	0.0000	0.0000
N1	0.0292 (8)	0.0251 (8)	0.0245 (7)	0.0136 (7)	0.0020 (6)	0.0044 (6)
N2	0.0236 (7)	0.0254 (8)	0.0236 (7)	0.0137 (6)	0.0016 (5)	0.0029 (6)
C1	0.0390 (11)	0.0333 (10)	0.0280 (9)	0.0189 (9)	0.0005 (8)	0.0072 (8)
C2	0.0499 (13)	0.0318 (11)	0.0277 (9)	0.0201 (10)	0.0074 (9)	0.0085 (8)
C3	0.0430 (12)	0.0235 (9)	0.0329 (9)	0.0139 (9)	0.0131 (8)	0.0057 (7)
C4	0.0311 (10)	0.0218 (9)	0.0335 (9)	0.0120 (8)	0.0091 (7)	0.0034 (7)
C5	0.0267 (10)	0.0268 (10)	0.0492 (12)	0.0096 (8)	0.0100 (9)	0.0042 (9)
C6	0.0234 (9)	0.0317 (11)	0.0500 (12)	0.0120 (9)	0.0012 (8)	0.0028 (9)
C7	0.0254 (9)	0.0265 (9)	0.0345 (9)	0.0139 (8)	−0.0007 (7)	−0.0012 (7)
C8	0.0294 (10)	0.0387 (11)	0.0374 (10)	0.0201 (9)	−0.0070 (8)	−0.0022 (8)
C9	0.0357 (11)	0.0455 (12)	0.0287 (9)	0.0265 (10)	−0.0011 (8)	0.0059 (8)
C10	0.0298 (10)	0.0331 (10)	0.0271 (8)	0.0180 (8)	0.0034 (7)	0.0072 (7)
C11	0.0242 (8)	0.0217 (8)	0.0260 (8)	0.0126 (7)	0.0019 (6)	0.0002 (6)
C12	0.0263 (9)	0.0196 (8)	0.0265 (8)	0.0113 (7)	0.0043 (7)	0.0018 (6)
Ni2	0.0182 (1)	0.0182 (1)	0.0196 (2)	0.0091 (1)	0.0000	0.0000
N3	0.0191 (7)	0.0209 (7)	0.0215 (6)	0.0087 (6)	0.0010 (5)	0.0000 (5)
N4	0.0224 (7)	0.0215 (7)	0.0212 (6)	0.0124 (6)	0.0011 (5)	0.0001 (5)
C13	0.0230 (8)	0.0282 (9)	0.0238 (8)	0.0120 (7)	0.0024 (6)	−0.0005 (7)
C14	0.0242 (9)	0.0344 (10)	0.0256 (8)	0.0116 (8)	0.0046 (7)	0.0041 (7)
C15	0.0230 (9)	0.0284 (9)	0.0291 (9)	0.0091 (7)	0.0033 (7)	0.0081 (7)
C16	0.0207 (8)	0.0235 (9)	0.0294 (8)	0.0094 (7)	−0.0012 (7)	0.0027 (7)
C17	0.0297 (10)	0.0208 (9)	0.0359 (10)	0.0095 (8)	−0.0002 (8)	0.0037 (7)
C18	0.0335 (10)	0.0189 (8)	0.0370 (10)	0.0131 (8)	−0.0022 (8)	−0.0029 (7)
C19	0.0280 (9)	0.0227 (8)	0.0283 (8)	0.0151 (7)	−0.0022 (7)	−0.0018 (7)
C20	0.0387 (11)	0.0288 (9)	0.0280 (9)	0.0217 (9)	−0.0017 (8)	−0.0046 (7)
C21	0.0397 (11)	0.0328 (10)	0.0258 (8)	0.0228 (9)	0.0059 (8)	−0.0004 (7)
C22	0.0302 (9)	0.0268 (9)	0.0265 (8)	0.0164 (8)	0.0067 (7)	0.0036 (7)
C23	0.0204 (8)	0.0205 (8)	0.0226 (7)	0.0117 (7)	−0.0004 (6)	0.0007 (6)
C24	0.0182 (7)	0.0218 (8)	0.0217 (7)	0.0096 (7)	−0.0012 (6)	0.0011 (6)
O2W	0.17 (3)	0.13 (2)	0.070 (8)	0.064 (14)	0.029 (11)	0.057 (10)
O3W	0.103 (10)	0.047 (6)	0.096 (9)	0.042 (7)	−0.066 (8)	−0.042 (6)
Ag1	0.0301 (1)	0.0415 (1)	0.0319 (1)	0.0163 (1)	−0.0012 (1)	−0.0025 (1)
N5	0.084 (2)	0.088 (2)	0.0441 (12)	0.0616 (18)	−0.0165 (12)	−0.0209 (13)
N6	0.0533 (13)	0.0660 (15)	0.0356 (10)	0.0366 (12)	−0.0068 (9)	−0.0062 (10)
C25	0.0431 (13)	0.0557 (15)	0.0367 (11)	0.0292 (12)	−0.0066 (9)	−0.0044 (10)
C26	0.0337 (11)	0.0452 (13)	0.0316 (10)	0.0194 (10)	−0.0034 (8)	0.0003 (9)
O1	0.095 (2)	0.0522 (14)	0.154 (3)	0.0465 (15)	0.0142 (19)	0.0261 (16)
N7	0.0363 (11)	0.0363 (11)	0.052 (2)	0.0182 (5)	0.0000	0.0000
O1WA	0.077 (2)	0.0626 (17)	0.0815 (19)	0.0403 (16)	0.0047 (17)	0.0071 (15)
O1WB	0.060 (9)	0.066 (10)	0.070 (10)	0.027 (8)	−0.005 (8)	−0.023 (8)

### Geometric parameters (Å, °)

**Table:**

Ag1—C25	2.043 (3)	C6—C7	1.443 (3)
Ag1—C26	2.055 (3)	C7—C8	1.401 (3)
Ni1—N1^i^	2.090 (2)	C7—C11	1.399 (3)
Ni1—N2^i^	2.101 (2)	C8—C9	1.370 (4)
Ni1—N1^ii^	2.0903 (19)	C9—C10	1.395 (4)
Ni1—N2^ii^	2.1014 (16)	C11—C12	1.437 (3)
Ni1—N1	2.0903 (17)	C1—H1	0.9500
Ni1—N2	2.1014 (18)	C2—H2	0.9500
Ni2—N4^iii^	2.090 (2)	C3—H3	0.9500
Ni2—N4	2.0898 (16)	C5—H5	0.9500
Ni2—N4^iv^	2.090 (2)	C6—H6	0.9500
Ni2—N3	2.0925 (15)	C8—H8	0.9500
Ni2—N3^iii^	2.0925 (19)	C9—H9	0.9500
Ni2—N3^iv^	2.092 (2)	C10—H10	0.9500
O2W—O2W^v^	1.27 (5)	C13—C14	1.396 (3)
O2W—O2W^vi^	1.27 (4)	C14—C15	1.375 (3)
O2W—O3W	1.24 (3)	C15—C16	1.409 (3)
O1—N7	1.204 (3)	C16—C17	1.433 (3)
N1—C1	1.336 (3)	C16—C24	1.399 (3)
N1—C12	1.354 (3)	C17—C18	1.346 (3)
N2—C11	1.358 (3)	C18—C19	1.435 (3)
N2—C10	1.333 (2)	C19—C20	1.404 (3)
N3—C24	1.355 (2)	C19—C23	1.406 (3)
N3—C13	1.331 (3)	C20—C21	1.367 (3)
N4—C23	1.354 (2)	C21—C22	1.395 (3)
N4—C22	1.333 (3)	C23—C24	1.438 (3)
N5—C25	1.125 (4)	C13—H13	0.9500
N6—C26	1.142 (4)	C14—H14	0.9500
C1—C2	1.398 (4)	C15—H15	0.9500
C2—C3	1.364 (4)	C17—H17	0.9500
C3—C4	1.409 (3)	C18—H18	0.9500
C4—C5	1.427 (4)	C20—H20	0.9500
C4—C12	1.404 (3)	C21—H21	0.9500
C5—C6	1.338 (3)	C22—H22	0.9500
			
Ag1···Ni2	4.7375 (3)	O1WA···N6	2.911 (4)
Ag1···Ni1^v^	4.7580 (3)	O1WA···O1WA^viii^	2.924 (5)
Ag1···Ni2	4.7375 (3)	O1WB···N6	2.860 (18)
Ag1···Ni2	4.7375 (3)	O2W···N7	3.265 (15)
Ni1···Ag1^vii^	4.7580 (3)	O2W···N7	3.265 (15)
Ni1···Ag1^iv^	4.7580 (3)	O2W···N7	3.265 (15)
Ni1···Ag1^vi^	4.7580 (3)	O2W···O3W^vi^	2.36 (3)
Ni2···Ag1^iii^	4.7375 (2)	O3W···O3W^v^	2.80 (3)
Ni2···Ag1^iv^	4.7375 (3)	O3W···O3W^vi^	2.80 (2)
Ni2···Ag1	4.7375 (3)		
			
C25—Ag1—C26	176.78 (13)	N2—C10—C9	122.9 (2)
N1—Ni1—N2	79.41 (7)	N2—C11—C12	116.93 (19)
N1—Ni1—N2^i^	97.46 (7)	N2—C11—C7	123.20 (17)
N1—Ni1—N1^ii^	91.91 (7)	C7—C11—C12	119.87 (19)
N1—Ni1—N2^ii^	167.39 (9)	C4—C12—C11	119.3 (2)
N1^i^—Ni1—N2	167.39 (8)	N1—C12—C11	117.42 (18)
N2—Ni1—N2^i^	92.56 (7)	N1—C12—C4	123.30 (18)
N1^ii^—Ni1—N2	97.46 (9)	C2—C1—H1	119.00
N1—Ni1—N1^i^	91.91 (8)	N1—C1—H1	119.00
N1^i^—Ni1—N2^i^	79.41 (8)	C3—C2—H2	120.00
N1^i^—Ni1—N1^ii^	91.91 (9)	C1—C2—H2	120.00
N1^i^—Ni1—N2^ii^	97.46 (9)	C2—C3—H3	120.00
N1^ii^—Ni1—N2^i^	167.39 (10)	C4—C3—H3	120.00
N2^i^—Ni1—N2^ii^	92.56 (8)	C6—C5—H5	119.00
N1^ii^—Ni1—N2^ii^	79.41 (7)	C4—C5—H5	119.00
N2—Ni1—N2^ii^	92.56 (8)	C7—C6—H6	120.00
N3—Ni2—N4^iii^	94.73 (7)	C5—C6—H6	120.00
N4—Ni2—N4^iv^	92.55 (7)	C9—C8—H8	120.00
N3^iii^—Ni2—N4	169.48 (8)	C7—C8—H8	120.00
N4—Ni2—N4^iii^	92.55 (7)	C8—C9—H9	120.00
N3^iv^—Ni2—N4^iv^	79.56 (7)	C10—C9—H9	120.00
N3^iv^—Ni2—N3^iii^	94.05 (8)	C9—C10—H10	118.00
N3^iv^—Ni2—N4^iii^	169.48 (7)	N2—C10—H10	119.00
N3^iv^—Ni2—N4	94.73 (7)	N3—C13—C14	122.84 (18)
N4^iv^—Ni2—N4^iii^	92.55 (7)	C13—C14—C15	118.97 (19)
N3^iii^—Ni2—N4^iii^	79.56 (8)	C14—C15—C16	119.77 (18)
N3—Ni2—N4	79.56 (6)	C15—C16—C17	123.61 (18)
N3—Ni2—N3^iv^	94.05 (7)	C15—C16—C24	116.88 (18)
N3—Ni2—N4^iv^	169.48 (8)	C17—C16—C24	119.51 (18)
N3—Ni2—N3^iii^	94.05 (6)	C16—C17—C18	120.94 (18)
N3^iii^—Ni2—N4^iv^	94.73 (7)	C17—C18—C19	121.10 (18)
O2W^v^—O2W—O2W^vi^	60 (3)	C20—C19—C23	117.26 (17)
O2W^v^—O2W—O3W	140.5 (17)	C18—C19—C23	119.06 (18)
O2W^vi^—O2W—O3W	109 (3)	C18—C19—C20	123.68 (18)
Ni1—N1—C1	128.62 (17)	C19—C20—C21	119.42 (19)
C1—N1—C12	117.80 (19)	C20—C21—C22	119.6 (2)
Ni1—N1—C12	113.07 (12)	N4—C22—C21	122.61 (18)
Ni1—N2—C10	129.53 (16)	N4—C23—C24	117.32 (16)
Ni1—N2—C11	112.89 (12)	C19—C23—C24	119.65 (16)
C10—N2—C11	117.54 (19)	N4—C23—C19	123.03 (18)
Ni2—N3—C24	113.01 (12)	N3—C24—C23	117.01 (15)
C13—N3—C24	118.01 (16)	N3—C24—C16	123.43 (18)
Ni2—N3—C13	128.98 (13)	C16—C24—C23	119.56 (16)
C22—N4—C23	118.00 (16)	N3—C13—H13	119.00
Ni2—N4—C23	112.93 (12)	C14—C13—H13	118.00
Ni2—N4—C22	128.90 (13)	C15—C14—H14	120.00
O1^v^—N7—O1^vi^	119.6 (2)	C13—C14—H14	121.00
O1—N7—O1^vi^	119.6 (2)	C14—C15—H15	120.00
O1—N7—O1^v^	119.6 (3)	C16—C15—H15	120.00
N1—C1—C2	122.6 (2)	C16—C17—H17	120.00
C1—C2—C3	119.6 (2)	C18—C17—H17	119.00
C2—C3—C4	119.4 (2)	C19—C18—H18	119.00
C3—C4—C12	117.2 (2)	C17—C18—H18	119.00
C3—C4—C5	123.5 (2)	C21—C20—H20	120.00
C5—C4—C12	119.31 (19)	C19—C20—H20	120.00
C4—C5—C6	121.5 (2)	C22—C21—H21	120.00
C5—C6—C7	120.8 (2)	C20—C21—H21	120.00
C6—C7—C8	123.5 (2)	N4—C22—H22	119.00
C8—C7—C11	117.5 (2)	C21—C22—H22	119.00
C6—C7—C11	118.95 (18)	Ag1—C25—N5	177.4 (3)
C7—C8—C9	119.3 (2)	Ag1—C26—N6	177.3 (3)
C8—C9—C10	119.5 (2)		
			
N2—Ni1—N1—C1	−175.0 (2)	Ni2—N4—C23—C19	174.54 (17)
N2—Ni1—N1—C12	−3.53 (14)	Ni2—N4—C23—C24	−4.6 (2)
N1^i^—Ni1—N1—C1	14.2 (2)	C22—N4—C23—C24	179.7 (2)
N1^i^—Ni1—N1—C12	−174.32 (15)	N1—C1—C2—C3	2.4 (4)
N2^i^—Ni1—N1—C1	93.8 (2)	C1—C2—C3—C4	0.0 (4)
N2^i^—Ni1—N1—C12	−94.76 (15)	C2—C3—C4—C5	177.9 (2)
N1^ii^—Ni1—N1—C1	−77.8 (2)	C2—C3—C4—C12	−2.4 (3)
N1^ii^—Ni1—N1—C12	93.70 (15)	C3—C4—C12—N1	2.8 (3)
N1—Ni1—N2—C10	−176.73 (19)	C3—C4—C12—C11	−176.24 (19)
N1—Ni1—N2—C11	0.57 (13)	C5—C4—C12—N1	−177.5 (2)
N2^i^—Ni1—N2—C10	−79.62 (19)	C3—C4—C5—C6	−178.9 (2)
N2^i^—Ni1—N2—C11	97.68 (14)	C12—C4—C5—C6	1.4 (3)
N1^ii^—Ni1—N2—C10	92.71 (18)	C5—C4—C12—C11	3.5 (3)
N1^ii^—Ni1—N2—C11	−89.99 (14)	C4—C5—C6—C7	−3.4 (4)
N2^ii^—Ni1—N2—C10	13.06 (19)	C5—C6—C7—C8	−178.1 (2)
N2^ii^—Ni1—N2—C11	−169.63 (14)	C5—C6—C7—C11	0.4 (3)
N3—Ni2—N4—C23	2.76 (14)	C11—C7—C8—C9	−3.6 (3)
N3^iv^—Ni2—N4—C22	−88.9 (2)	C6—C7—C11—N2	−175.3 (2)
N3^iv^—Ni2—N4—C23	96.01 (15)	C6—C7—C8—C9	174.9 (2)
N4^iv^—Ni2—N4—C22	−9.1 (2)	C6—C7—C11—C12	4.5 (3)
N4^iv^—Ni2—N4—C23	175.74 (15)	C8—C7—C11—N2	3.3 (3)
N4^iii^—Ni2—N4—C22	83.6 (2)	C8—C7—C11—C12	−176.9 (2)
N4—Ni2—N3—C13	178.8 (2)	C7—C8—C9—C10	1.1 (4)
N4—Ni2—N3—C24	−0.55 (14)	C8—C9—C10—N2	2.3 (4)
N3^iv^—Ni2—N3—C13	84.70 (19)	C7—C11—C12—N1	174.50 (19)
N3^iv^—Ni2—N3—C24	−94.62 (15)	C7—C11—C12—C4	−6.4 (3)
N3^iii^—Ni2—N3—C13	−9.7 (2)	N2—C11—C12—C4	173.41 (18)
N3^iii^—Ni2—N3—C24	171.03 (15)	N2—C11—C12—N1	−5.7 (3)
N4^iii^—Ni2—N3—C13	−89.50 (19)	N3—C13—C14—C15	−1.6 (4)
N4^iii^—Ni2—N3—C24	91.19 (15)	C13—C14—C15—C16	−1.5 (4)
N3—Ni2—N4—C22	177.9 (2)	C14—C15—C16—C17	−175.9 (2)
N4^iii^—Ni2—N4—C23	−91.59 (15)	C14—C15—C16—C24	3.3 (3)
C12—N1—C1—C2	−2.1 (3)	C15—C16—C17—C18	177.9 (2)
Ni1—N1—C12—C4	−173.11 (16)	C24—C16—C17—C18	−1.2 (4)
Ni1—N1—C1—C2	169.10 (17)	C15—C16—C24—N3	−2.3 (3)
C1—N1—C12—C4	−0.6 (3)	C15—C16—C24—C23	178.2 (2)
C1—N1—C12—C11	178.46 (19)	C17—C16—C24—N3	176.9 (2)
Ni1—N1—C12—C11	6.0 (2)	C17—C16—C24—C23	−2.6 (3)
Ni1—N2—C10—C9	174.54 (17)	C16—C17—C18—C19	3.0 (4)
C11—N2—C10—C9	−2.7 (3)	C17—C18—C19—C20	178.6 (3)
Ni1—N2—C11—C7	−177.83 (16)	C17—C18—C19—C23	−0.9 (4)
Ni1—N2—C11—C12	2.4 (2)	C18—C19—C20—C21	−176.9 (2)
C10—N2—C11—C7	−0.2 (3)	C23—C19—C20—C21	2.6 (4)
C10—N2—C11—C12	−179.97 (18)	C18—C19—C23—N4	178.0 (2)
C13—N3—C24—C23	178.91 (19)	C18—C19—C23—C24	−2.9 (3)
Ni2—N3—C24—C16	178.82 (17)	C20—C19—C23—N4	−1.5 (3)
Ni2—N3—C13—C14	−176.67 (17)	C20—C19—C23—C24	177.6 (2)
C24—N3—C13—C14	2.6 (3)	C19—C20—C21—C22	−1.1 (4)
Ni2—N3—C24—C23	−1.7 (2)	C20—C21—C22—N4	−1.7 (4)
C13—N3—C24—C16	−0.6 (3)	N4—C23—C24—N3	4.3 (3)
C23—N4—C22—C21	2.8 (3)	N4—C23—C24—C16	−176.2 (2)
Ni2—N4—C22—C21	−172.11 (18)	C19—C23—C24—N3	−174.9 (2)
C22—N4—C23—C19	−1.2 (3)	C19—C23—C24—C16	4.7 (3)

Symmetry codes: (i) −*y*+1, *x*−*y*, *z*; (ii) −*x*+*y*+1, −*x*+1, *z*; (iii) −*x*+*y*, −*x*+1, *z*; (iv) −*y*+1, *x*−*y*+1, *z*; (v) −*y*, *x*−*y*, *z*; (vi) −*x*+*y*, −*x*, *z*; (vii) *x*+1, *y*, *z*; (viii) *y*, −*x*+*y*, −*z*.

### Hydrogen-bond geometry (Å, °)

**Table:**

*D*—H···*A*	*D*—H	H···*A*	*D*···*A*	*D*—H···*A*
C3—H3···N5^ix^	0.95	2.45	3.284 (4)	147
C5—H5···O1^vi^	0.95	2.36	3.176 (5)	144
C8—H8···O1WA^viii^	0.95	2.54	3.465 (4)	166
C17—H17···O1	0.95	2.47	3.423 (4)	177
C20—H20···N6^viii^	0.95	2.60	3.312 (3)	132

Symmetry codes: (ix) *y*−1/3, −*x*+*y*−2/3, −*z*+1/3; (vi) −*x*+*y*, −*x*, *z*; (viii) *y*, −*x*+*y*, −*z*.
